# Hypothermic sepsis in time since death estimation – a case report

**DOI:** 10.1007/s00414-024-03193-4

**Published:** 2024-02-20

**Authors:** Stefan Potente, Victoria Hanser, Sara Heinbuch, Arne Wrede, Nadine Schäfer, Peter Schmidt

**Affiliations:** 1 Department of Legal Medicine, University of Saarland Medical School, Homburg/Saar, Germany; 2 Department of Neuropathology, University of Saarland Medical School, Homburg/Saar, Germany

**Keywords:** Sepsis, Hypothermia, Death time estimation, Tricuspid valve endocarditis, Death time estimation, Prism-method

## Abstract

Both hyper- and hypothermia are problematic in temperature based forensic time since death estimation. Hyperthermia may occur in infection, traumatic brain injury, and intoxication. Hypothermia is encountered predominantly in exposure. Sepsis may present itself clinically as hypothermic. Sepsis is not uncommon in the forensic setting and mostly occurs in the context of malpractice accusations. There is usually little overlap between sepsis and typical forensic time since death estimation scenarios of violent or otherwise suspicious deaths. In the presented case, hypothermia and time since death estimations *did* collide. An inmate was found dead in his jail cell. Wardens claimed they had visually approached him alive relatively shortly prior. Rectal temperature measurements, using two separate crime scene thermometers as well as temperature loggers, revealed low rectal temperature at relatively high ambient temperature. These findings suggested a much longer postmortem interval and consequently raised doubts about the stated timeline. The wardens’ claims were however confirmed by camera recordings, which also allowed a reasonable estimate of the true time of death. The cause of death was confirmed as septic organ failure at autopsy, which explained low rectal temperature. The presence of Wischnewski-spots was noted. When the Prism-method was applied to the temperature recordings, low rectal temperature at the time of death was detected successfully. However, adaptation of the underlying equation for lower “starting temperature” did not produce satisfactory results. It is concluded that even though hypothermia at the time of death may possibly be detected from temperature data, attempts at time since death estimation for cases of hypothermia by adaptation of the equation should be avoided.

## Introduction

Forensic time since death (TsD) estimation in the early postmortem period is usually performed using temperature based methods [1]. Rectal temperature at the time of death (T_r@D_) is of outmost importance in this context. It is usually assumed as 37.2 °C and deviations may come in the form of hyperthermia or hypothermia at the time of death.

*Hyperthermia* causes in forensic medicine include traumatic brain injury, intoxication / medication, infection, stress, and others [2–4]. Consequently, indicators for elevated T_r@D_ may include a particularly high rectal temperature (T_r_) long after finding the body, disproportion of high T_r_ and qualities of rigor/lividity, autopsy findings indicating traumatic brain injury or infection, histology findings indicating infection or other relevant pathology, and toxicology findings for drugs or medication. The Prism-method [5] was shown to potentially detect elevated T_r@D_ from temperature data even when T_r_ has decreased well below 37.2 °C.

*Hypothermia* in forensic medicine is encountered primarily in the context of exposure. In terms of TsD estimation, Henssge notes, – clearly directed at exposure – that “in case of generalized hypothermia the possible errors are potentially much larger, as the extent of hypothermia still compatible with life is much larger than hyperthermia. When substantial hypothermia is suspected, it is recommended to abandon TsD estimation altogether” [6]. Body temperature alterations may as well occur clinically in critically ill patients [7]. Sepsis in particular may include both hyper- or hypothermia. Sepsis is neither rare in general nor in forensic autopsy cases. Tsokos underlined the forensic relevance of sepsis in respect to medical negligence, since 50% of all sepsis cases constitute a sequel of nosocomial infections. Even though individual findings may also be present in various non-infectious diseases, typical constellations of findings may “to a certain degree” be seen as characteristic for sepsis [8, 9].

Hypothermic sepsis is reported as rare and with worse prognosis than hyperthermic sepsis [10, 11]. More severe sepsis was positively associated with greater likelihood for hypothermia [12] and the possibility of a triage screening for hypothermia in sepsis has been explored [13]. It was argued that hypothermia in sepsis was often self limiting and transient [14]. Hypothermic patients, are reported as cathectic, with acquired disease [15].

Naturally, there is little overlap between primarily clinical cases of forensic relevance and violent or otherwise suspicious deaths which usually require TsD estimation. To our knowledge, no case of hypothermic sepsis with relevance to TsD estimation has so far been reported.

### Case

A 24 year old man with a history of petty crime and iv. drug use was jailed for insubordination according to § 230 of the German code of criminal procedure (StPO). In jail he received drug substitution therapy (L-Polamidon® / Levomethadonhydrochloride) and remained otherwise uncooperative. He did not complain of any major discomfort.

Prison wardens gave the following statment: The inmate was last seen alive on the day of his death when he was approached both verbally and visually at around 12:30 h to be offered food, which he rejected. He was approached again at around 15:00 h and found lifeless in his cell. He was subsequently relocated to the cell block hallway and cardiopulmonary resuscitation (CPR) was performed over the course of 35 min.

**Fig. 1:**
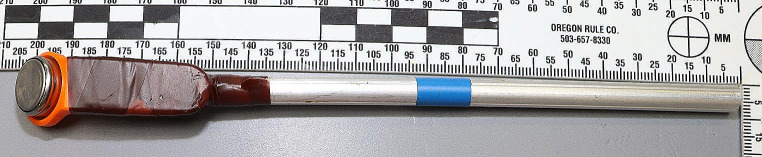
DS1922L data logger after removal of protective layers. Note blue marking at 120 mm distance to tip of logger assembly

Following CPR and the declaration of death at 15:35 h a T_r_ of 35.7 °C was measured by the emergency personel using a standard medical thermometer. No ambient temperature (T_a_ ) was taken at this point. On inspection by a police investigation team and a forensic pathologist at around 17:00 h, rigor mortis was not noticeable and patchy lividity was barely visible. T_a_ was measured as slightly above 23 °C inside the cell and slighlty below 23 °C in the hallway in front. T_r_ was measured as 30.0 °C at 17:00 h using the police’s crime scene thermometer. Immediate follow-up measurements using the forensic pathologist’s thermometer confirmed the measurements. Finally, one data logger (iButton DS1922L [16]), attached to a metal rod, was inserted into the rectum and another one was attached loosely to the foot using a long piece of string (see Fig. 1) to record T_a_. The wardens’ statements indicated a living individual at 2 h prior to ending CPR (T_r_ 35.7 °C) and 4.5 h prior to scene investigations (T_r_ 30.0 °C), in relatively high T_a_.

Application of the Nomogram method [1], executed for the parameters:

- time of measurement: 17:00 h
- T_r@D_ : 37.2 °C (standard)
- T_a_ : 23.0 °C
- T_r_ : 30.0 °C
- body weight: 59 kg

calculated the death time interval as 12.9 h before masurement ± 2.8 h (between 01:18 h and 06:54 h).

**Fig. 2:**
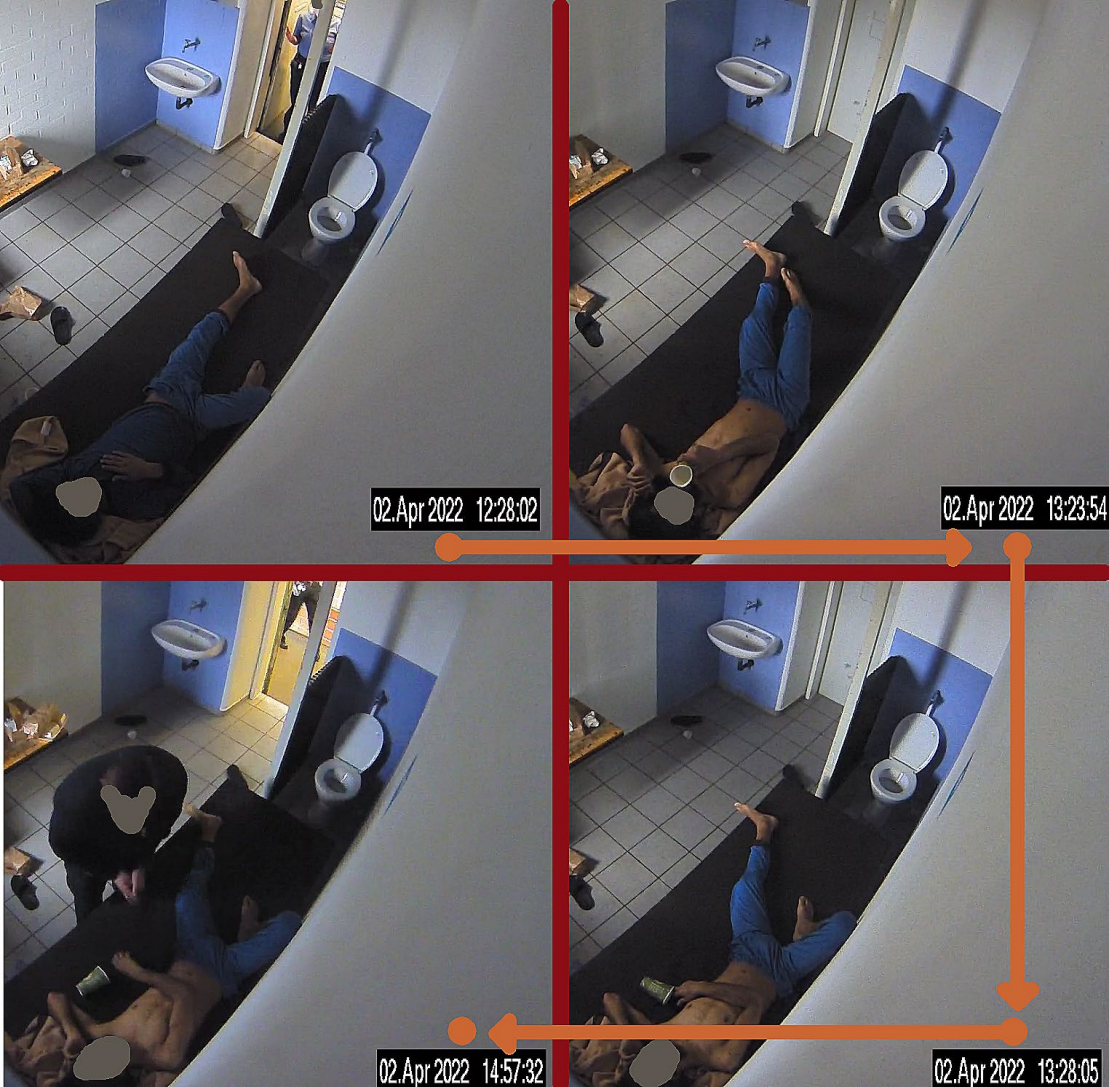
Screenshots from camera footage. Top left, 12:28 h: inmate is approached through the open cell door. Top right, 13:23 h: inmate has taken off his shirt and is seen drinking water from a cup. Lower right, 13:28 h: observable breathing seizes. Lower left, 14:57 h: inmate is found by wardens who initiate emergency response after checking pulse

Doubts about the wardens’ timeline were raised and it was speculated that proper confirmation of the inmate’s wellbeing had been skipped. However, the timeline was confirmed precisely by camera footage soon after. In addition, observable breathing as well as any body movement seized at 13:28 h during the recording (see Fig. 2).

Autopsy findings 2.5 days later included:

- young man of Middle Eastern descent
- no visible signs of assault
- firm rigor, faint lividity with an overall anaemic aspect of the skin and mucosa
- body weight of 59 kg (a reduction of 7 kg compared to admission 6 weeks prior)
- scars from past self inflicted injuries and injection of (most likely) drugs
- endocarditis of the tricuspid valve with adherent rubbery material (see Fig. 3, left)
  **Fig. 3:**
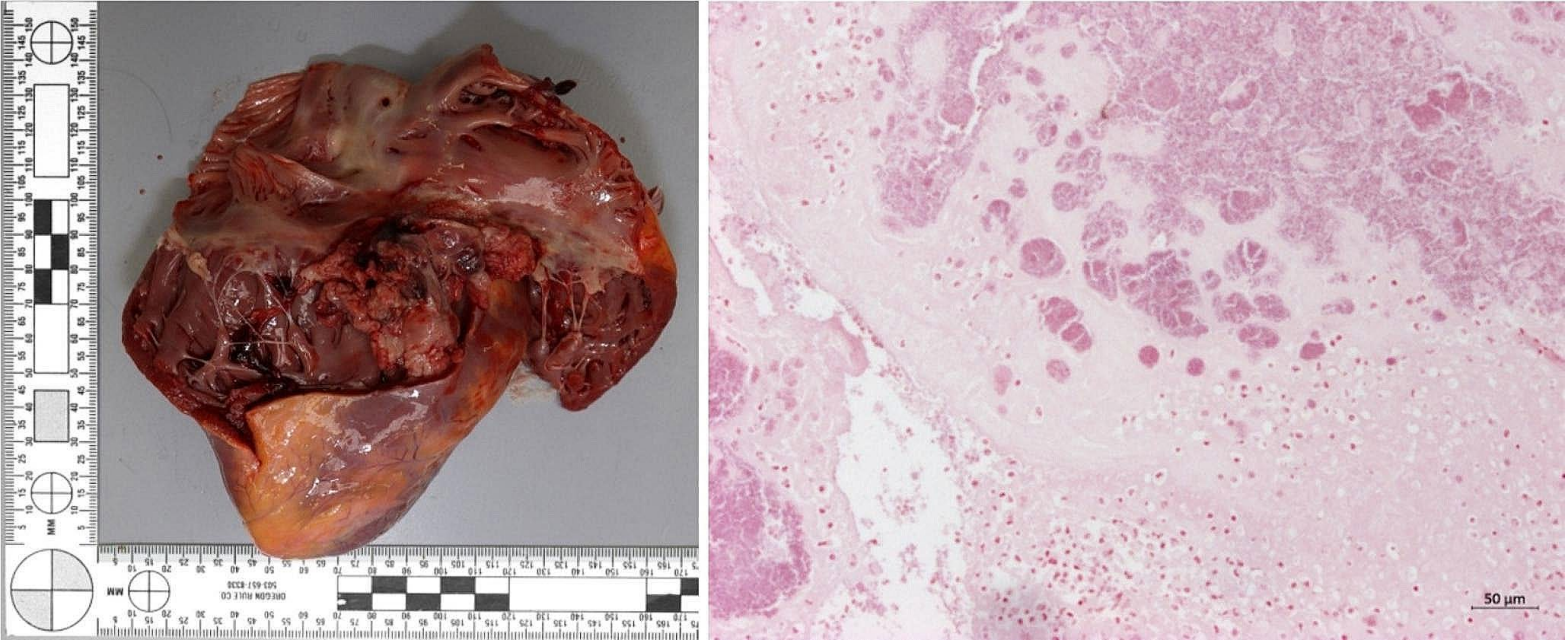
Vegetations on the tricuspid valve - left: gross inspection, right: histology (Gram stain, 100x magnification)
- bilateral pneumonia with lung abscesses and (on gross inspection) lung infarctions (see Fig. 4, left)
  **Fig. 4:**
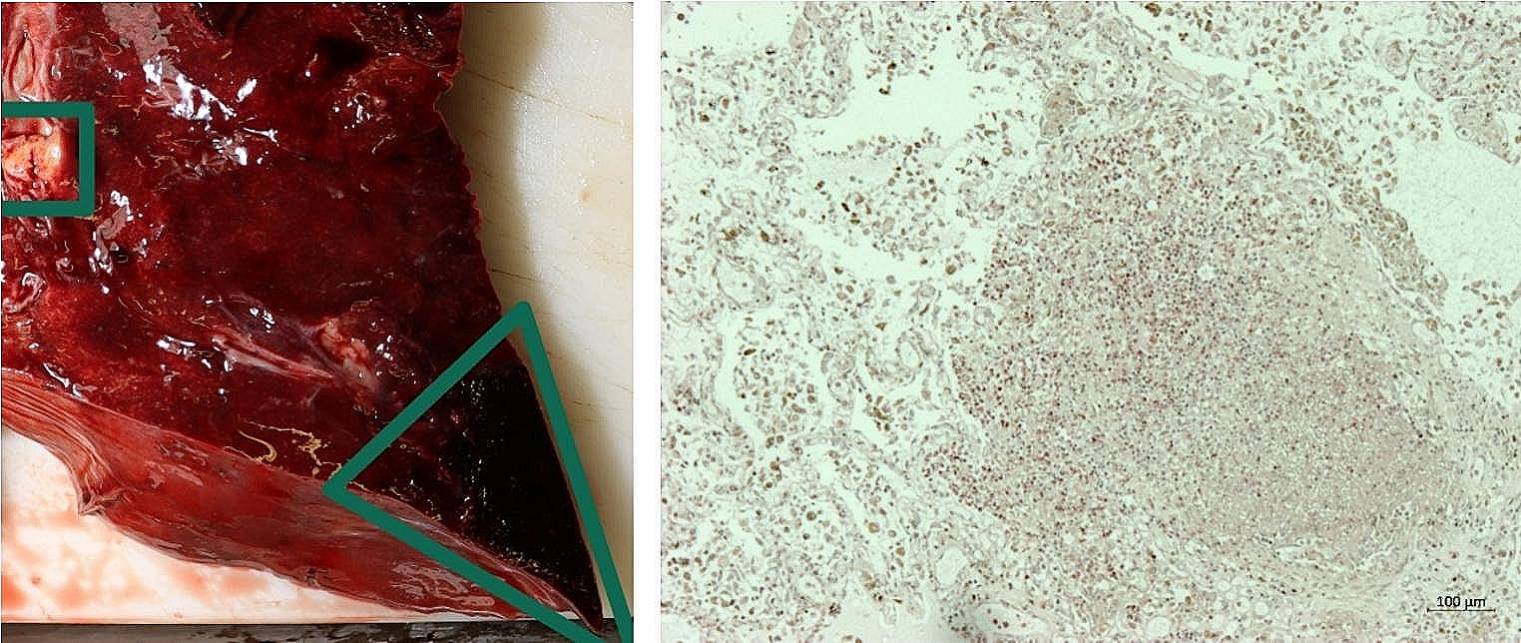
Lung abscess (square) and density/lung infarction (triangle) - left: gross inspection, right: histology (CAE stain, 50x magnification)
- petechial and patchy haemorrhages on the inner aspects of the lips and the intestines
- enlargement of the spleen (615 g)
- Wischnewski-spots of the stomach (see Fig. 5, left)
  **Fig. 5:**
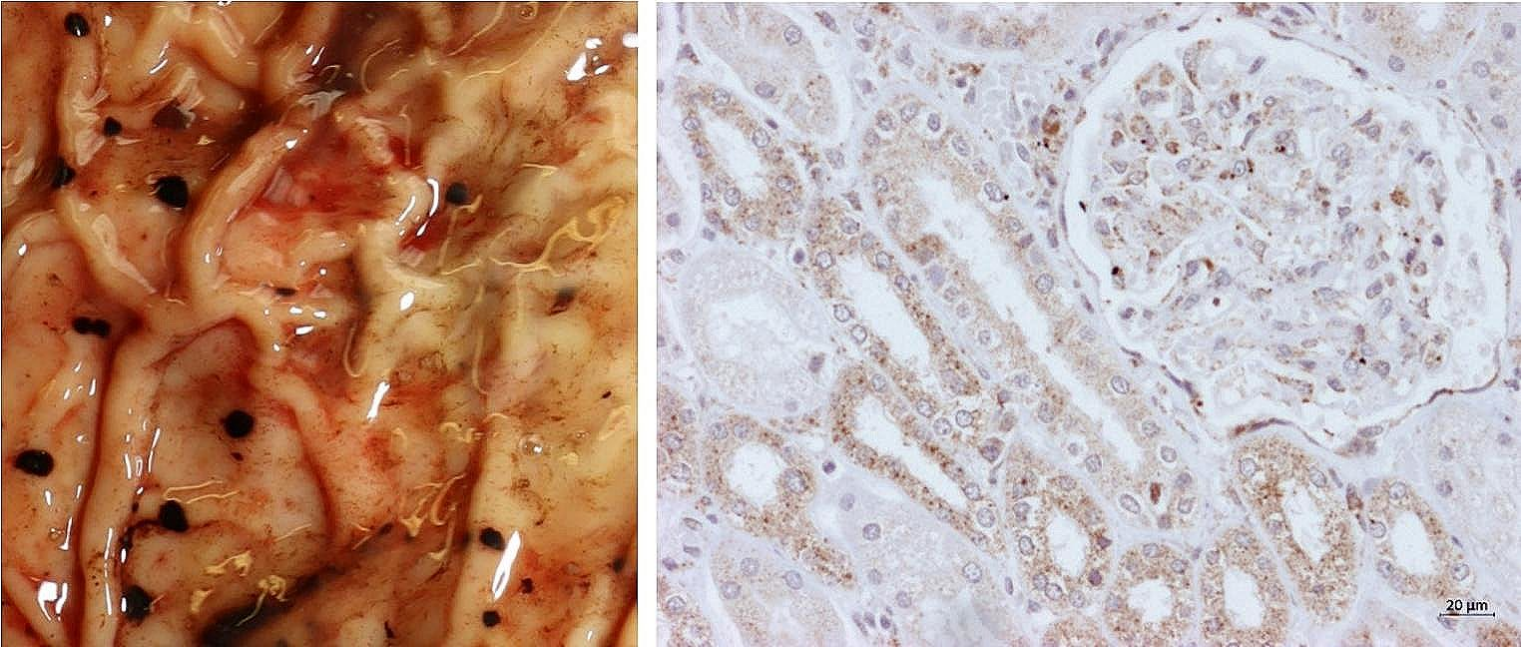
Left: Wischnewski-spots of the stomach, right: immunohistochemical staining, kidney (HSP 70, 200x magnification)

Cause of death was determined as septic organ failure with predominant failure of the lungs due to endocarditis of the tricuspid valve with septic dissememination and lung abcesses. No further autopsy findings indicative of hypothermia (frost erythema, red lividity, “empty scrotum”, pancreatic haemorrhage, iliopsoas muscle haemorrhage [17]) were observed.

In histology the following findings were observed:

- destructive endocarditis of the triscuspid valve with intensive bacterial growth (see Fig. 3, right)
- septic foci in the myocardium and lungs (see Fig. 4, right)
- bacterial aggregates in blood vessels
- foreign body granulomas in the lungs

Immunohistochemical staining for heat shock protein 70 (HSP 70) [18] in the kidney showed moderate (+ 2) to severe (+ 3) color reaction in the tubule epithelium and grade + 3 staining of the glomeruli, partly including Bowman’s capsule (classification in [19], see Fig. 5, right). Oil red lipid staining of pancreas, heart, and kidney was negative.

Toxicology findings confirmed drug substitution therapy within therapeutic limits and therapeutic levels for ibuprofen, but were otherwise unremarkable (see Table 1).

**Table 1:** Toxicology findings from venous blood (*) 2-ethylidine-1,5-dimethyl-3,3diphenyl-1-pyrrolidine

Substance	Concentration	Notes
Methadone EDDP(*)	0.11 mg/l ≈ 0.025 mg/l	Methadone metabolite
Quetiapine	≈ 0.12 mg/l	GC/MS
Ibuprofen	positive	

**Fig. 6:**
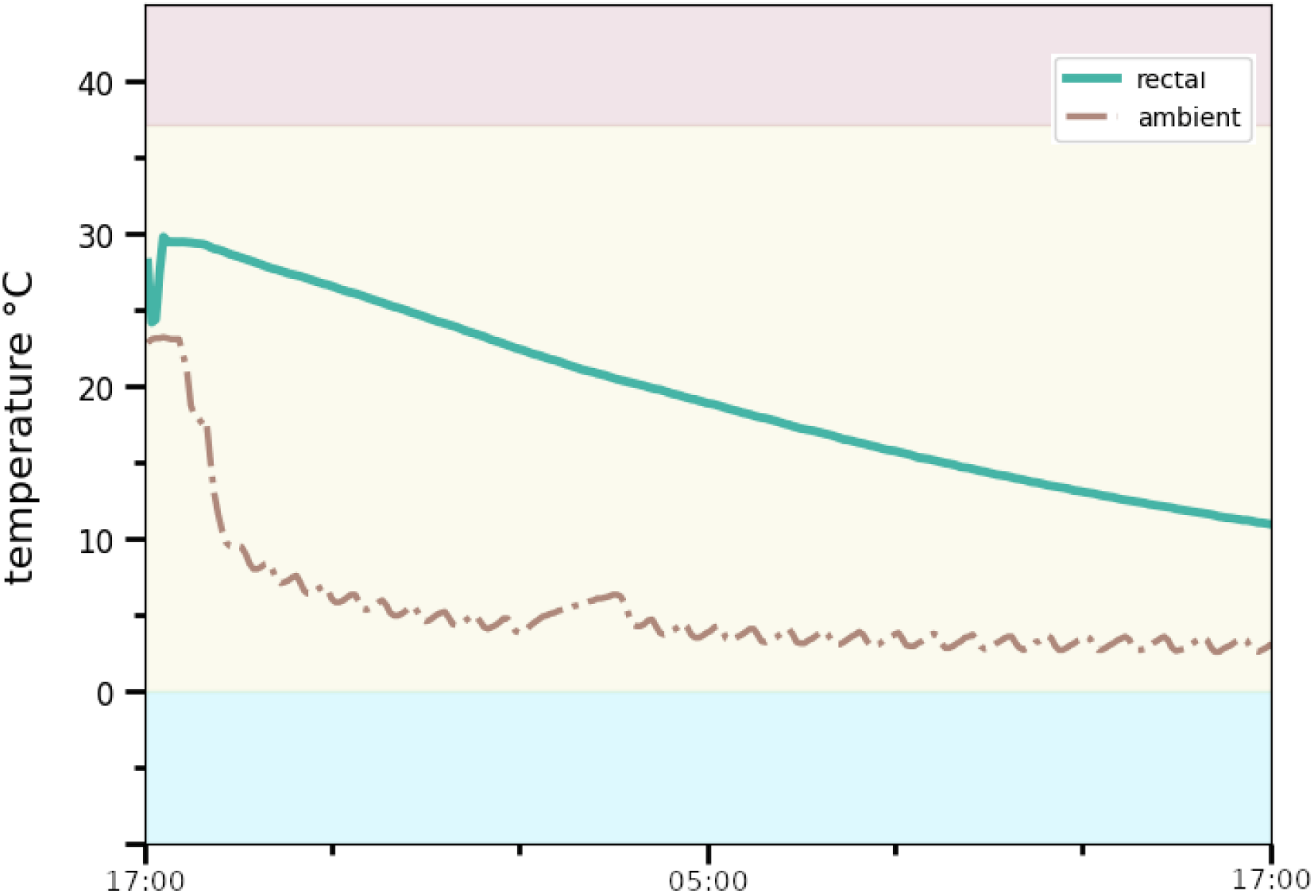
iButton DS1922L logger data: T_r_ (green) and T_a_ (dashed red) over time, starting at 17:00 h. Water freezing point (0 °C) and standard body temperature (37.2 °C) colored for reference

**Fig. 7:**
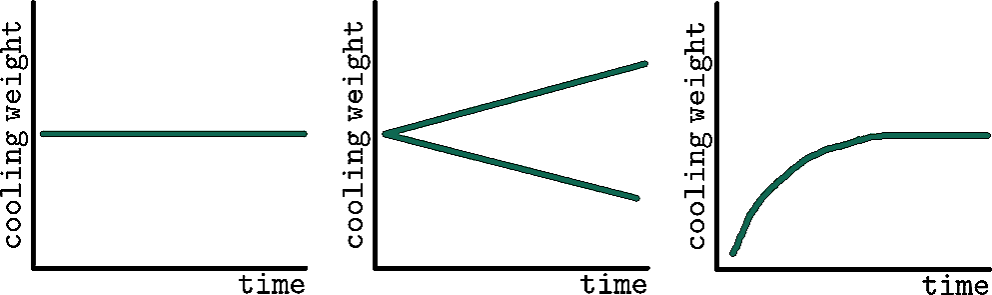
Basic CWC-shapes in Prism. Left: correct parameters indicated by constant cooling weight. Center: false-high or false-low assumed T_a_ leads to increase or decrease of cooling weight over time. Right: elevated Tr_@D_ produces false-low CWC in early cooling stages

For TsD estimation, the so called prism-method was applied to temperature data from loggers (see Fig. 6). The method utilizes serial cooling data. Using brute force calculation, the cooling weight (”the weight according to which the body cools” as a result of body geometry, body weight and external factors) between two distinct points on the cooling curve is calculated. Multiple such cooling weights are then plotted as a cooling weight curve (CWC). CWC allow the analysis of parameter sets, in particular assumptions for T_r@D_, T_a,_ and the cooling weight. Ideally, CWC may be used to identify the correct parameter set to use in definite TsD estimation. Different CWC shapes have been identified in the past (correct parameter sets, false low and high assumed T_a_, elevated T_r@D_, see Fig. 7). The method’s principles were laid out in detail in [5, 20].

**Fig. 8:**
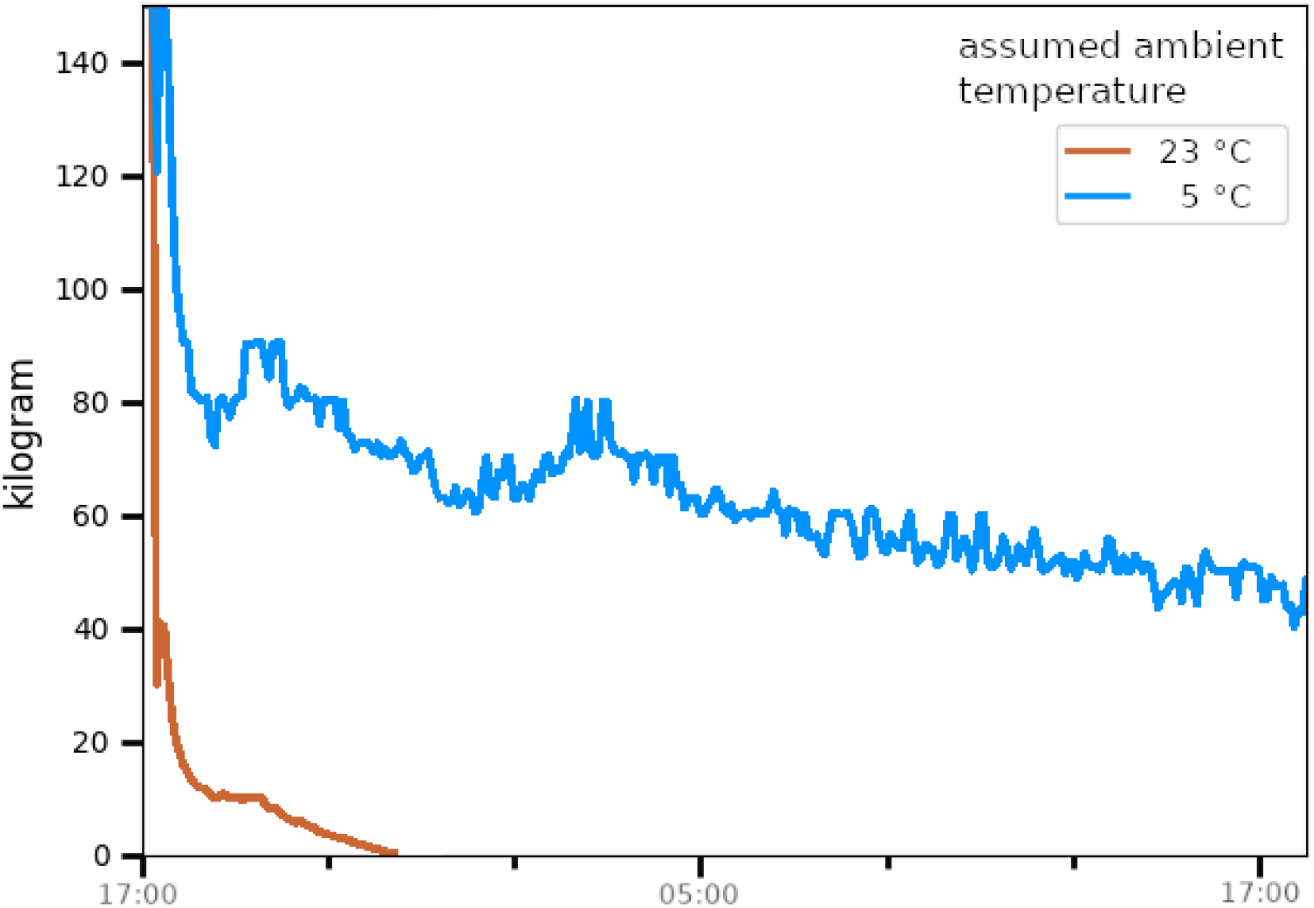
Prism cooling weight curves (CWC) for assumed T_a_ of 23.0 °C (red) and 5.0 °C (blue)

For the case at hand, CWC were calculated for assumed T_a_ of 23 °C (jail) and T_a_ of 5 °C (cooling facilities after removal of the body), as depicted in Fig. 8: for 23 °C, the CWC “drops out” shortly after removal of the body from the scene, since no body weight, regardless how small, represents the cooling curve assuming high T_a_. The assumed T_a_ of 5 °C produced a slight downward slope, indicating that 5 °C is assumed still too high.

However, both the T_a_ assumptions produced extremely high cooling weights in the initial phase of recording. This pattern is reverse to the CWC which indicates elevated T_r@D_, where temperature falls too steeply in the early stages of cooling beyond 37.2 °C (“as if extremely light”). In reduced T_r@D_ the body cools too slow (“as if extremely heavy”) relative to T_a_.

It was then attempted to compensate for the established hypothermia via adjustment of the Nomogram method’s equation. Such adjustment cannot be performed within the Nomogram itself, since T_r@D_ is hard-coded into its geometry (see [21] for details).

However, some electronic calculation aids, such as dedicated spreadsheets, scripts, and Schweitzer’s online solution [22], allow the adjustment of assumed T_r@D_. By lowering T_r@D_, as low as 34 °C, the calculated TsD interval was in fact shifted “further into the future”. However, those alternative intervals, even using the most extreme assumptions, did not even come close to the established likely time of death of 13:28 h (see Table 2).

**Table 2:** TsD estimation results for various assumed T_r@D_ (37.2 °C standard). Time of measurement 17:00 h, 59 kg body weight, 22.9 °C T_a_

T_r@D_	From…	Until…
37.2 °C	01:18 h	06:54 h
36.0 °C	02:36 h	08:12 h
35.0 °C	03:42 h	09:18 h
34.0 °C	05:00 h	10:36 h

## Discussion

The case’s autopsy findings and case history were typical for sepsis and tricuspid valve endocarditis, comparable to similar published cases [23–26].

The case is nevertheless special. It combines severe sepsis outside of the hospital setting, hypothermia, and a focus on body temperature when TsD was of genuine forensic interest. Hypothermic sepsis has not yet been published in the forensic literature. Documentation was good. The first T_r_ reading by emergency personel should in our opinion be dismissed, since the thermometer was unsuitable for use in the dead. Measurements by crime scene thermometer were double checked and followed by the continual recording of T_r_ and T_a_. Autopsy, histology and toxicology were performed and police investigations further substantiated the case.

The application of the Prism-method did support the hypothesis of antemortem hypothermia due to sepsis. However, lowering T_r@D_ in the underlying equation after Henssge did not result in correct TsD estimation. Possibly, a different mathematical frame work is needed for cases, where death is part of an already ongoing cooling process and much less of a “starting point” than in the regular model. The presence of Wischnewski-spots is an interesting side aspect of our case. Their pathogenesis is not yet fully understood. They are usually found in exposure, but have also been discussed for diabetic ketoacidosis, alcohol, drugs, pesticide poisoning, and even fatal burns [27–31]. Potentially, the understanding of such findings in hypothermic sepsis may contribute to the understanding of Wischnewski-spots in general.

## Conclusion and outlook

Hypotheremic sepsis is a rare case to be considered outside of the hospital setting. It can substantially complicate TsD estimation. Attempts at compensating for hypothermia were unsuccessfull. The use of temperature loggers and the Prism-method should however be considered in crime scene work. It supported the assumption of hypothermic sepsis, which should be used as an indicator to abandon TsD estimation for the case.

## Data Availability

Not applicable
